# Editorial: Similarities and differences between cellular and molecular mechanisms of normal brain aging and neurodegeneration

**DOI:** 10.3389/fnagi.2025.1602391

**Published:** 2025-04-16

**Authors:** Anamaria Jurcau

**Affiliations:** Department of Psycho-Neurosciences and Rehabilitation, University of Oradea, Oradea, Romania

**Keywords:** brain aging, neurodegeneration, neuroinflammation, hypoxia, neurovascular coupling

The increase in human life expectancy over the last century in the majority of the world has come at the cost of an increased incidence of neurodegenerative diseases, of which dementias are the most feared ones, and for which age is the most prominent risk factor (Azam et al., 2021). Although the underlying causes are poorly understood, aging is a universal phenomenon in living organisms and leads to a decline in the function of all organs and systems. Extensive research has identified 12 hallmarks of aging (genomic instability, telomere attrition, epigenetic alterations, loss of proteostasis, disabled macroautophagy, deregulated nutrient sensing, mitochondrial dysfunction, cellular senescence, stem cell exhaustion, alterations in intercellular communication, chronic inflammation, and gut dysbiosis) that are strongly interconnected (López-Otín et al., 2023), and have also been identified in aging brains. However, substantial variation in the progression of age-related alterations in brain structure and function has been observed among individuals across species, accounting for brain resilience, brain reserve, and cognitive compensation (Stern et al., 2023). Given the complexity of aging in the brain, this Research Topic in the *Frontiers in Aging Neuroscience* aims to identify the differences between the mechanisms that contribute to normal brain aging as opposed to neurodegeneration at both the cellular and molecular levels.

First, in their original research, Cao et al. show that chronic exposure to hypoxia leads to changes in protein expression with increased expression of caspase-3 mRNA and protein levels (caspase-3 is a key regulator of neuronal apoptosis) and of a number of genes that lead to accelerated cellular senescence, such as p16, p21, and p53. These changes occurred primarily in the prefrontal cortex and hippocampus of the mice studied, and were associated with a decrease in the gray matter volume of the left piriform cortex, caudate nucleus and left visceral area, in addition to altered functional connectivity between the basal ganglia, hippocampus, and anterior limbic cortex despite a compensatory increase in the diameter of both common carotid arteries and the left internal carotid artery.

The importance of proper brain oxygenation is also emphasized by Jiao in his mini-review, which explains how hypoxic pockets—transient cortical areas of oxygen depletion where oxygen supply no longer matches metabolic demand for milliseconds to seconds—are due to disruptions in capillary perfusion or increased local metabolic demand during intense neural activity. Although these sporadically occurring hypoxic pockets activate angiogenesis and neurovascular remodeling, their effects tend to accumulate and can progressively impair neural function, mainly in the prefrontal cortex and hippocampus, igniting neurodegeneration.

In their submitted perspective, Jia and Shen draw attention to the contribution of chronic inflammation to neurodegeneration, an area of active research (Jurcau et al., 2024). The term “inflammaging” refers to a persistent low-grade inflammation characteristically found in aging organisms and linked to damaged or malfunctioning cells and to aberrant immune responses (Soraci et al., 2024). Mitigating inflammation has been shown to delay aging successfully. One such strategy is the use of platelet factor 4 (or platelet-derived exerkine CXCL4), which increases in the plasma following exercise or under the influence of the longevity factor klotho (Park et al., 2023) and reduces inflammation by activating the Janus kinase (JAK)/signal transducers and activators of transcription (STAT) pathway (Buka et al., 2024).

In their review, Komleva et al. outline the challenges that need to be overcome in developing “aging clocks” that are able to show significant deviations from the normal aging trajectory and identify individuals with accelerated brain aging at risk for cognitive impairment. They emphasize the non-linear changes in gene expression in midlife, which makes the study of methylation of CpG subgroups in DNA, along with the expression of genes that increase the susceptibility to impaired brain bioenergetics, especially important in establishing the biological age of an individual. However, a number of other factors, such as inflammatory biomarkers, or altered metabolic signaling, should be integrated into these predictive tools. As such, assessing the brain aging gap becomes increasingly more complex, having to integrate cognitive tests, neuroimaging techniques, serological biomarkers, and genetic tests, a task in which artificial intelligence, or the digital twin technology may assist in a more personalized approach (Lehman et al., 2024).

Nonetheless, because rejuvenating therapeutic approaches are still in their infancy (Nunkoo et al., 2024) and have serious side effects, all manuscripts submitted to this Research Topic emphasize that to date the only proven methods to date that can help in achieving healthy brain aging are a healthy diet, even with periods of caloric restriction, physical exercise, and memory training, which act in synergy to delay brain aging, improve neurovascular coupling and increase synaptic plasticity (Bennett et al., 2024).
